# Astrocyte Infection during Rabies Encephalitis Depends on the Virus Strain and Infection Route as Demonstrated by Novel Quantitative 3D Analysis of Cell Tropism

**DOI:** 10.3390/cells9020412

**Published:** 2020-02-11

**Authors:** Madlin Potratz, Luca Zaeck, Michael Christen, Verena te Kamp, Antonia Klein, Tobias Nolden, Conrad M. Freuling, Thomas Müller, Stefan Finke

**Affiliations:** 1 Friedrich-Loeffler-Institut (FLI), Federal Research Institute for Animal Health, Institute of Molecular Virology and Cell Biology, 17493 Greifswald-Insel Riems, Germany; Madlin.Potratz@fli.de (M.P.); Luca.Zaeck@fli.de (L.Z.); m.christen1994@gmail.com (M.C.); Antonia.Klein@fli.de (A.K.); Conrad.Freuling@fli.de (C.M.F.); Thomas.Mueller@fli.de (T.M.); 2 Thescon GmbH, 48653 Coesfeld, Germany; verena_tekamp@gmx.de; 3 ViraTherapeutics GmbH, 6020 Innsbruck, Austria; tobias.nolden@boehringer-ingelheim.com

**Keywords:** rabies, uDISCO, 3D imaging, rabies pathogenicity, astrocyte infection

## Abstract

Although conventional immunohistochemistry for neurotropic rabies virus (RABV) usually shows high preference for neurons, non-neuronal cells are also potential targets, and abortive astrocyte infection is considered a main trigger of innate immunity in the CNS. While in vitro studies indicated differences between field and less virulent lab-adapted RABVs, a systematic, quantitative comparison of astrocyte tropism in vivo is lacking. Here, solvent-based tissue clearing was used to measure RABV cell tropism in infected brains. Immunofluorescence analysis of 1 mm-thick tissue slices enabled 3D-segmentation and quantification of astrocyte and neuron infection frequencies. Comparison of three highly virulent field virus clones from fox, dog, and raccoon with three lab-adapted strains revealed remarkable differences in the ability to infect astrocytes in vivo. While all viruses and infection routes led to neuron infection frequencies between 7–19%, striking differences appeared for astrocytes. Whereas astrocyte infection by field viruses was detected independent of the inoculation route (8–27%), only one lab-adapted strain infected astrocytes route-dependently [0% after intramuscular (i.m.) and 13% after intracerebral (i.c.) inoculation]. Two lab-adapted vaccine viruses lacked astrocyte infection altogether (0%, i.c. and i.m.). This suggests a model in which the ability to establish productive astrocyte infection in vivo functionally distinguishes field and attenuated lab RABV strains.

## 1. Introduction

The rabies virus (RABV) is a highly neurotropic virus, which inevitably causes lethal disease in mammals after onset of neurological signs [1]. As a non-segmented, single-stranded RNA virus of negative RNA polarity, RABV belongs to the *Rhabdoviridae* family in the order *Mononegavirales* [2]. With nucleoprotein N, phosphoprotein P, matrix protein M, glycoprotein G, and the large polymerase L, the 12 kb genome of RABV encodes five virus proteins, all of which are essential for virus replication and spread [3]. In addition to essential roles of the virus proteins in genome replication and virus assembly, multiple accessory functions of the RABV proteins have been identified. RABV pathogenicity has mainly been attributed to a potent interference with the innate immune system by N, P, and M [4,5,6,7,8,9,10], and neuronal survival regulation by G [11,12,13,14]. Most pathogenicity studies, however, were performed on already attenuated virus backbones. Thus, differences in their ability to cause disease between highly virulent field virus isolates and lab-adapted, less pathogenic RABV strains are poorly understood. Moreover, it is unclear how molecular differences identified in virulent and attenuated viruses affect virus replication and spread in the infected animal and how the complex virus–host interplay eventually results in either disease or an abortive infection.

In vivo, after infection of neurons, RABV spreads trans-synaptically from infected to connected neurons [15]. Retrograde axonal transport of RABV over long distances [16,17] along microtubules [18,19] is a key step in RABV neuroinvasion and is essential for infection of the central nervous system (CNS) through the peripheral nervous system. Co-internalization together with the neuronal p75NTR (tumor necrosis factor receptor superfamily member 16; TNFRSF16) receptor, subsequent retrograde axonal transport of RABV particles in endocytic vesicles, and post-replicative anterograde axonal transport of newly formed RABV have been visualized by live virus particle tracking in sensory neurons [20,21], emphasizing the capacity of hijacking neuron-specific machineries for long distance transport to synaptic membranes. However, internalization and axonal transport of lab-adapted viruses [20,21] together with the use of vaccine virus vectors for trans-synaptic tracing [22,23] demonstrate that the general capacity of axonal transport and trans-synaptic spread cannot explain mechanistic differences between highly virulent RABV and more attenuated lab strains. Differences between RABV lab strains in the efficiency of trans-synaptic spread [24] indicate that the efficacy of the involved processes, more than the capability itself, may contribute to RABV spread in vivo.

With nAChR (nicotinic acetylcholine receptor), NCAM (neuronal cell adhesion molecule), p75NTR, and mGluR2 (metabotropic glutamate receptor subtype 2) supporting RABV entry [25,26,27,28], several RABV receptors have been discussed. However, none of these receptors are essential for CNS infection by RABV, and a broad panel of non-neuronal cell types can be infected in vitro [29], indicating that cell tropism of RABV is not restricted to neurons by receptor specificity.

Most in vivo studies report a strict neurotropic infection. Directly after exposure by bite, however, muscle cells are infected (reviewed in [30]), and infection of non-neuronal cells in the CNS can occur [29,31,32]. Use of recombinant Cre recombinase-expressing RABV led to the identification of abortively infected glial cells in infected mouse brains, strongly suggesting infection of and virus elimination from these cells by a potent type I interferon response [33]. Accordingly, abortive infection of non-neuronal cells and induction of innate immune responses may play an important role in the infection process itself and in regulating downstream adaptive immune pathways. Indeed, a model based on in vitro-infected astrocytes suggests that, in contrast to wild-type RABV, attenuated RABV activates inflammatory responses in astrocytes through increased double-strand RNA (dsRNA) synthesis and recognition by retinoic acid-inducible gene I (RIG-I)/melanoma differentiation-associated protein 5 (MDA5) [34]. Highly virulent field RABV isolates are able to evade or at least delay host immune reactions [35], which may allow virus replication to reach pathogenic levels, whereas early innate immune induction via astrocytes or other glial cells by attenuated viruses does not. Nevertheless, all productive RABV infections eventually cause rabies as a disease, which is always associated with an encephalitis. However, differences appear to exist as field viruses cause less tissue damage and neuroinflammation than attenuated lab RABV [34,36,37].

To compare the cell tropism of highly virulent field and lab-adapted RABVs in the CNS, we employed the novel immunostaining-compatible tissue clearing technique uDISCO (ultimate 3D imaging of solvent-cleared organs) [38,39,40] for detection and quantification of RABV infection in neurons and astrocytes in solvent-cleared brain tissue. After confocal laser scan acquisition of large confocal z-stacks in thick tissue slices, three-dimensional (3D) reconstructions were performed to visualize the cellular context of RABV infection. Frequencies of RABV infection in neurons and astrocytes were determined after intramuscular (i.m.) and intracerebral (i.c.) inoculation, leading to novel insights regarding the ability to establish RABV infection in non-neuronal astrocytes, its correlation with RABV virulence, and its dependence on the infection route. These data strongly support a model in which, contrary to in vitro conditions where all viruses are able to infect non-neuronal astrocytes, there are differences between field and lab-adapted viruses in their ability to replicate to detectable levels in astrocytes in vivo. The infection of astrocytes with virulent field RABV and the accumulation of interferon antagonistic virus proteins may block rapid innate immune induction, thereby contributing to immune evasion, even in the context of glial cell infection.

## 2. Materials and Methods

### 2.1. Cell Lines, Primary Brain Cell Preparation, and Cultivation

Mouse neuroblastoma cells (Na42/13) were used for virus amplification. All cells were provided by the Collection of Cell Lines in Veterinary Medicine (CCLV), Friedrich-Loeffler-Institut, Insel Riems, Germany. Primary neurons and astrocytes were prepared from 1-day-old neonatal Sprague Dawley rats (P0–P1) [41]. The rats were decapitated, the heads were disinfected in 70% ethanol, the brains were removed, and the hippocampi were separated and mechanically minced. The hippocampal cells were transferred into ice-cold Hank´s Balanced Salt Solution (HBSS) and stored on ice. After preparation, the brain cells were dissociated by adding 0.25% trypsin + EDTA (Gibco; Thermo Fisher Scientific, Darmstadt, Germany) and DNase I (Applichem, Darmstadt, Germany) and incubated for 15 min at 37 °C. Afterwards, further dissociation in Neurobasal-A Medium (NB-A, Gibco; Thermo Fisher Scientific) was performed by using a glass Pasteur pipette with a fire-polished tip. The cells were purified with an Optiprep^TM^ gradient (with concentrations from bottom to the top: 17.3%, 12.4%, 9.9%, and 7.4%) and centrifuged for 15 min at 800× *g* without brake. Next, the cells were washed with NB-A medium and counted with the LUNA-II Automated Cell Counter (Logos Biosystems, Villeneuve d’Ascq, France). The neuronal cells were seeded on coverslips in a 24-well plate and cultured for two weeks at 37 °C and 5% CO_2_ in serum-free NB-A supplemented with 2% B27 (Gibco; Thermo Fisher Scientific), 1% GlutaMAX (Gibco; Thermo Fisher Scientific), 1% Penicillin-Streptomycin (stock: 10,000 U/mL penicillin and 10 mg/mL streptomycin; Sigma-Aldrich, Taufkirchen, Germany), and 0.2% Gentamicin (stock: 50 mg/mL; Gibco; Thermo Fisher Scientific) until they were used for infection experiments.

### 2.2. Viruses

RABV isolates used in this study comprised five recombinant virus clones and one non-recombinant lab virus. The recombinant field virus clones rRABV Dog and rRABV Fox have been described before [42]. SAD L16 is a recombinant virus clone of attenuated vaccine virus SAD B19 live vaccine virus [43]. The Evelyn Rokitnicki Abelseth (ERA) strain [44] is a progenitor virus strain of live vaccine virus SAD B19 [45] and was obtained from the FLI virus archive (FLI ID N 12829).

A cDNA full length clone (pRABV Rac) from a raccoon RABV isolate (Alabama, USA 1991; FLI archive ID N 13205) [46] was generated by full length RT-PCR amplification of the 12 kb cDNA genome with the primer pair 5′-TCGATCCCGGGTCACGCTTAACAACAAAA-3′/5′-TAATACACCTGCCCATGCCGACCCACGCTTAACAAAAAAACAA-3′. After PCR amplification of a 2.7 kb vector DNA fragment from pCMV HaHd ampR Br 322 ori with the primers 5′-TCTGTTTGCTTGATGGTTTTTTTTGTCTTTGTTGTTTTTTTGTTAAGCGTGGGTCGGCATGGCATCTCCAC-3′ and 5′-TTTTTGTAGATGATACTGTCTACTTCTTCTCTGATTTTGTTGTTAAGCGTGACCCGGGACTCCGGGTTTCGTC-3′, the fragments were combined by linear to linear recombination, and the resultant recombinant rRABV Rac virus was rescued and amplified according to previously described protocols [42]. The sequence of the full-length cDNA clone was deposited at GenBank (accession no. MN862283).

A CVS-11 (Challenge Virus Street-11; sequence accession no. LT839616) cDNA full length clone (pCVS-11) was generated by RT-PCR amplification of 7.5kb and 3.7 kb cDNA fragments from CVS-11 RNA with the primers pairs 5′-AGTTTCAGACGTCTCAGTC-3′/5′-CTAGTAGGGATGATCTAGATC-3′ and 5′-GACTGAGACGTCTGAAACT-3′/5′ CATTGCAGATAGGATAGAG-3′, respectively. The resultant fragments were assembled in combination with EcoRI-linearized 3.4 kb vector plasmid pCVS-11-termini by Hot Fusion reaction [47]. Plasmid pCVS-11-termini was generated by PCR amplification of a 2.7 kb DNA fragment from pCMV HaHd ampR Br 322 ori [42] with the primers 5′-GACCCGGGACTCCGGGTTTC-3′ and 5′-GGGTCGGCATGGCATCTCCA-3′, and Hot Fusion assembly with a synthetic DNA fragment containing the CVS-11 (accession no. LT839616) regions from nucleotide positions 1–580 (3′-genome end) and 11759–11927 (5′-genome end) separated by a non-viral EcoRI restriction site. The full-length cDNA clone was 99.99% identical to CVS-11 GenBank sequence no. LT839616 (1 nt mismatch in the N gene at nucleotide position 1263, which was already present in #LT839616 as a minor SNP (single nucleotide polymorphism), resulting in the amino acid exchange E398V). Recombinant rCVS-11 was rescued in Na42/13 neuroblastoma cells by co-transfection of pCVS-11, pCAGGS-based expression plasmids for RABV N, P, and L [48], and a pCAGGS-T7Pol vector comprising a codon-optimized bacteriophage T7 RNA polymerase gene. Three to six days after transfection, the supernatants were transferred to new Na42/13 cells. Two days after the transfer, infectious virus was identified by N and G protein-specific indirect immunofluorescence.

rRABV Dog, rRABV Fox, rRABV Rac, and rCVS-11 were amplified on Na42/13 cells. BSR T7/5 cells [49] were used for amplification of SAD L16 and ERA viruses. Infectious virus titers in cell culture supernatants were determined by end point dilution and titration on Na42/13 cells.

### 2.3. Antibodies

To verify the presence of RABV, a polyclonal rabbit serum against recombinant RABV P protein (P160-5, immunofluorescence 1:5000, uDISCO 1:3000) and a polyclonal goat serum against RABV N (goat anti-RV N, immunofluorescence 1:4000), which have been described previously [40,50], were used. The polyclonal chicken anti-glial fibrillary acidic protein (GFAP) antibody (Thermo Fisher Scientific, Darmstadt, Germany; #PA1-10004, uDISCO 1:1500), the polyclonal rabbit anti-GFAP (Dako, #Z0334, immunofluorescence 1:500), the polyclonal guinea pig anti-NeuN antibody (Synaptic Systems, Goettingen, Germany; #266004, uDISCO 1:800), and the rabbit anti-MAP2 antibody (Abcam, Cambridge, UK; #ab32454, immunofluorescence 1:250) were purchased from their respective suppliers.

### 2.4. Infection of in Vitro Cell Cultures and Immunofluorescence Staining

Two-week-old primary rat hippocampal cell cultures were infected with 1 × 10^3^ infectious units of rRABV Dog, rRABV Fox, rCVS-11, and SAD L16, and cultivated for 24 h at 37 °C and 5% CO_2_. Indirect immunofluorescence was performed by standard techniques after fixation with 4% paraformaldehyde (PFA) in phosphate-buffered saline (PBS) for 30 min and 15 min permeabilization with 0.5% Triton X-100 in PBS. Afterwards, samples were blocked with 0.025% skim milk powder in PBS for 15 min. Immunostainings were executed by 1.5 h incubation with primary antibodies, three wash steps with PBS, followed by 1 h incubation with secondary antibodies and additional Hoechst33342 (1 µg/mL) for staining of the nuclear chromatin. Specimens were mounted on coverslips and were analyzed by confocal laser scanning microscopy.

### 2.5. Brain Samples and Mouse Infections

Three- to four-week-old BALB/c mice (Charles-River, Germany) were infected with rRABV Rac, rCVS-11, ERA, and SAD L16 viruses using two different inoculation routes and two different viral doses, essentially as described before [51]. Two groups of six animals each were anesthetized and infected i.m. with 10^2^ or 10^5^ TCID_50_/30 µL, and an additional group of three mice was infected i.c. with 10^2^ TCID_50_/30 µL. The weight and the clinical score, ranging from zero to four, of all mice were observed for 21 days post infection (dpi). When reaching a clinical score of two or three (ruffled fur, slowed movement, weight loss >15%), the animals were anaesthetized with isoflurane and euthanized through cervical dislocation. Samples were taken, fixed with 4% paraformaldehyde (PFA) for one week, and stored for further processing. All remaining animals were euthanized at 21 dpi. Mouse experimental studies on the characterization of lyssaviruses were evaluated by the responsible animal care, use, and ethics committee of the State Office for Agriculture, Food Safety, and Fishery in Mecklenburg-Western Pomerania (LALFF M-V) and gained approval with permissions 7221.3–2–001/18.

### 2.6. Archived Mouse Brains Infected with rRABV Dog and rRABV Fox

To minimize animal experiments, archived PFA-fixed brains from previous pathogenicity trials [42] were used for the analysis of rRABV Dog and rRABV Fox virus infections in mice. Similar to the mouse experiments with rRABV Rac, rCVS-11, ERA, and SAD L16 described above, mice were inoculated via the i.c. or the i.m. route.

### 2.7. Ultimate 3D Imaging of Solvent-Cleared Organs (uDISCO)

The clearing of brain tissue slices was performed as described previously [40] in modification of earlier publications [38,39]. Briefly, the PFA-fixed tissues were sectioned into 1 mm-thick slices using a vibratome (Leica VT1200S; Leica Biosystems, Wetzlar, Germany). All subsequent incubations steps were performed with gentle oscillation. To increase antibody diffusion and reduce tissue autofluorescence [38], the sections were pretreated with increasing concentrations of methanol (20%, 40%, 60%, 80%, and twice in 100%; dilutions with distilled water, incubation for 1 h each) and bleached by overnight incubation at 4 °C with 5% H_2_O_2_ in 100% methanol. After removal of the bleaching solution, the samples were rehydrated with decreasing concentrations of methanol (80%, 60%, 40%, and 20%; dilutions with distilled water, incubation for 1 h each) and a subsequent wash with PBS for 1 h. To permeabilize the samples, they were washed twice for 1 h each with 0.2% Triton X-100 in PBS and subsequently incubated for 48 h at 37 °C in 0.2% Triton X-100/20% DMSO/0.3 M glycine in PBS. The samples were then blocked by incubation with 0.2% Triton X-100/10% DMSO/6% donkey serum in PBS for 48 h at 37 °C. Primary antibodies were diluted in 3% donkey serum/5% DMSO in PTwH (0.2% Tween-20 in PBS with 10 µg/mL heparin), and incubation of samples was performed at 37 °C for 5 days. The antibody solution was refreshed after 2.5 days. The samples were washed with PTwH by exchanging the solution four times during the course of the day and subsequently incubated overnight in PTwH. Incubation with secondary antibodies was performed in 3% donkey serum in PTwH for 5 days at 37 °C, refreshing the secondary antibody solution once after 2.5 days. Subsequent washing was performed as described above following primary antibody incubation.

For tissue clearing, the samples were dehydrated with a series of *tert*-butanol (TBA) solutions (30%, 50%, 70%, 80%, 90%, and 96%; dilutions with distilled water, incubation for 2 h each), leaving 96% TBA on overnight. Following further dehydration in 100% TBA for 2 h, the samples were cleared in BABB-D15 [39] [1:2 mixture of benzyl alcohol (BA) and benzyl benzoate (BB), which is mixed with diphenyl ether (DPE) at a ratio of 15:1 and supplemented with 0.4 vol% DL-α-tocopherol] until they were optically transparent (2–6 h).

For confocal laser scanning microscopy, the samples were mounted in 3D-printed imaging chambers (printer: Ultimaker 2 + [Ultimaker, Utrecht, Netherlands], material: co-polyester, nozzle: 0.25 mm, layer height: 0.06 mm, wall thickness: 0.88 mm, wall count: 4, infill: 100%, no support structure; the corresponding .STL file is provided in the Supplementary Materials of Zaeck et al. [40]).

### 2.8. Confocal Laser Scanning Microscopy and Image Processing

The immunofluorescent staining of primary brain cells and infected tissues was visualized with a confocal laser scanning microscope (Leica DMI 6000 TCS SP5; Leica Microsystems, Wetzlar, Germany) equipped with a long free working distance 40× water immersion objective (NA = 1.1; Leica, #15506360). For image processing, a Dell Precision 7920 workstation was used (CPU: Intel Xeon Gold 5118, GPU: Nvidia Quadro P5000, RAM: 128 GB 2666 MHz DDR4, SSD: 2 TB; Dell, Frankfurt am Main, Germany). 

For quantification of infected cells in thick tissue sections, the image was split into individual channels using Fiji, an ImageJ (v1.52h) distribution package [52]. A bleach correction was performed (simple ratio; background intensity: 5.0), and brightness and contrast were adjusted for each channel. Objects were identified and counted with the 3D Objects Counter plugin [53]. The resulting objects map was overlaid with the RABV P channel to quantify infected objects. For each sample, at least six regions were imaged and analyzed. The 3D projections in Figure 2d,e were generated with Icy [54]. All other maximum z- and 3D projections, including the Supplementary Videos S1–S4, were generated with Fiji.

### 2.9. Statistical Analysis

Statistical significance was determined using two-way ANOVA followed by Tukey’s multiple comparison test using GraphPad Prism 7.05. 

## 3. Results

### 3.1. Astrocytes Are Readily Infected by both Field Viruses and Lab Strains in Mixed Primary Brain Cell Cultures

**Figure 1:**
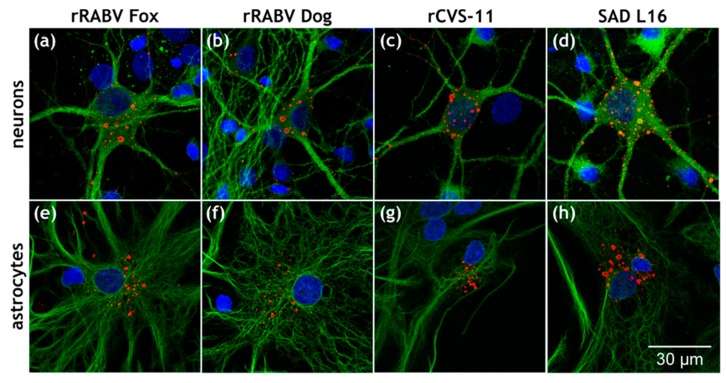
In vitro infection of primary hippocampus neurons and astrocytes by field and lab rabies virus (RABV). (**a**–**d**) Indirect immunofluorescence detection of RABV nucleoprotein N (red) and microtubule-associated protein 2 (MAP2) (green). Nuclei were counterstained with Hoechst33342 (blue). (**e**–**f**) Indirect immunofluorescence detection of RABV nucleoprotein N (red) and glial fibrillary acidic protein (GFAP) (green). Nuclei were counterstained with Hoechst33342 (blue). Negative controls for RABV detection are provided in Supplementary Figure S1.

To investigate whether field and lab-adapted RABVs differ in their ability to infect primary neurons and non-neuronal astrocytes, hippocampal brain cells were prepared from neonatal rats and were cultivated as mixed cultures containing neurons and glial cells. After 13 days of cultivation, the cultures were infected with 10^3^ infectious units of the recombinant field viruses rRABV Dog and rRABV Fox, and the lab-adapted strains rCVS-11 and SAD L16. After 24 h of infection, the cells were fixed and analyzed by confocal laser scanning microscopy using indirect immunofluorescence stainings against RABV nucleoprotein N, neuron marker MAP2 (microtubule-associated protein 2) and astrocyte marker GFAP (glial fibrillary acidic protein). All viruses infected both MAP2-positive neurons and GFAP-positive astrocytes, as demonstrated by the formation of RABV-positive cytoplasmic inclusion bodies (Figure 1). These data indicated that there was no obvious difference between the tested field and the lab-adapted viruses in their ability to infect neurons and non-neuronal astrocytes.

Although all four viruses were able to infect GFAP-positive cells in the hippocampal cell cultures, the majority of the infected cells were MAP2-positive neurons. To quantitatively compare astrocyte infection between viruses, the number of RABV N-positive cells was counted for an area of 25 mm^2^, and the percentage of GFAP-positive and -negative RABV-infected cells was determined (Table 1). All four viruses resulted in infection of GFAP-positive cells at levels ranging from 4.9% to 6.7%. Although a higher percentage of GFAP-positive cells could not be excluded because a high number of cells could not be classified into either positive or negative for GFAP, these results indicated that all four viruses could readily establish an infection in cultivated astrocytes and did not substantially differ in their ability to do so.

### 3.2. Field RABV Infects Neurons and Astrocytes In Vivo as Demonstrated by High-Resolution 3D Analysis of Infected Brain Tissue

**Figure 2:**
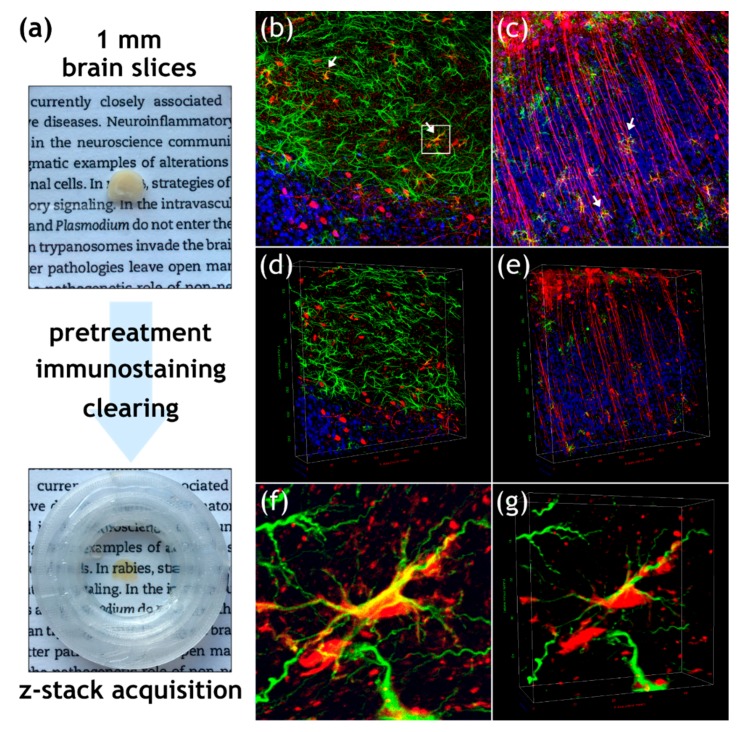
3D immunofluorescence imaging of field RABV-infected neurons and astrocytes in a mouse brain. (**a**) Workflow for 3D immunofluorescence imaging, including vibratome sectioning into 1 mm slices, pretreatment, immunostaining, and subsequent optical clearing with organic solvents. Confocal imaging and acquisition of cleared tissue slices was done in custom-made imaging containers (see lower image). (**b**,**c**) Maximum z- and (**d**–**e**) 3D projections of z-stack (x, y, z = 400 µm, 400 µm, 59 µm for D and 400 µm, 400 µm, 103 µm for E) after indirect immunofluorescence for RABV phosphoprotein P (red), GFAP (green), and NeuN (blue). White arrows in Figure 2b indicate RABV P and GFAP-positive astrocytes. (**f**) Maximum projection of detail from Figure 2b (see white box) with GFAP-positive cell (green) and associated RABV P fluorescence (red). (**g**) 3D projection of detail view from Figure 2f.

In order to test whether the primary cell culture infection experiments are comparable to the more complex in vivo situation, a sample preparation and imaging pipeline was established, which allows three-dimensional, high-resolution confocal laser scanning image acquisition from RABV-infected tissue for 3D reconstruction and quantification of infected cells. To this end, 1 mm-thick brain slices (Figure 2a) from clinically diseased rRABV Fox field virus-infected mice (i.m. infection route) were immunostained for RABV P, GFAP, and NeuN, optically cleared, and imaged. Acquisition of z-stacks by confocal laser scanning microscopy resulted in z-volumes of 400 µm × 400 µm × 50–100 µm (x, y, and z) from different areas of the infected brain. RABV-infected neurons of variable morphologies were detected by presence of NeuN and RABV P protein accumulation in neuronal cell bodies and neurites (Figure 2b–e; details in Supplementary Figure S2). Astrocytes were detected by filamentous GFAP-positive structures (Figure 2b–g).

Notably, besides accumulation of RABV P in NeuN-positive neurons, P also accumulated at GFAP-positive filaments (Figure 2b,c,f,g; white arrows), indicating a robust infection of astrocytes by field virus rRABV Fox. Evaluation of brain areas with differing localization patterns of the highlighted cellular subpopulations (Figure 2b,c) revealed that astrocyte infections were not restricted to particular regions of the brain but appeared in all imaged neuron layers or brain areas.

Because of the complex 3D morphology of both neurons (long axons in Figure 2b,c) and astrocytes (filamentous structure of GFAP signals in Figure 2f,g), 3D reconstruction of the imaged tissues (Figure 2d,e,g; Supplementary Videos S1 and S2) was performed in order to achieve reliable visualization and quantification of cells in downstream analyses.

### 3.3. Similar Levels of Field Virus Infection in both Neurons and Astrocytes in the Infected Mouse Brain

**Figure 3:**
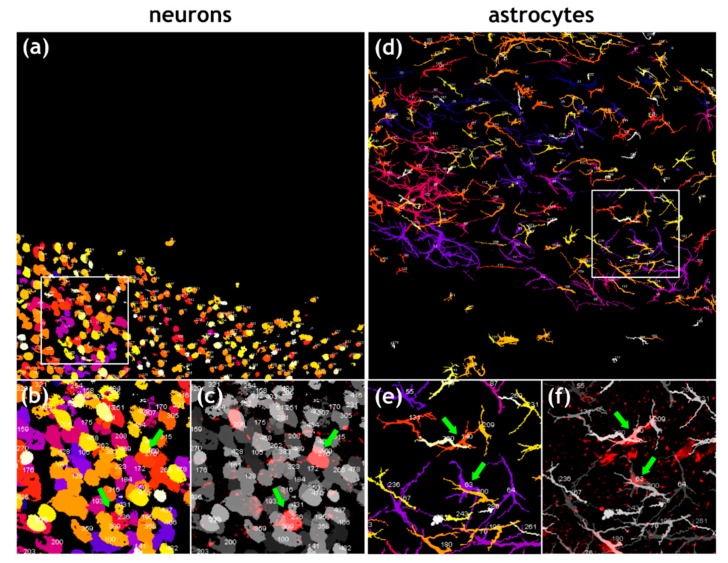
Quantification of RABV-infected neurons and astrocytes. (**a**) Maximum z-projection of objects map for NeuN-positive neurons generated from a confocal z-stack (see Figure 2b). Individual neurons in the z-stack were identified by NeuN-specific fluorescence and converted to objects. Numbers indicate individual cell counts (for improved legibility of the individual numbers, refer to the enlarged details in Supplementary Video S3). n = 762 neurons in a volume of 400 µm × 400 µm × 59 µm. The object colors indicate different z-positions (darker colors in the back and brighter colors in the front). (**b**) Detail of area indicated by white box in Figure 3a. Green arrows indicate RABV-positive neuron cell bodies. (**c**) Overlay of objects map (greyscale) with RABV P signals allows identification and counting of rRABV Fox-infected neurons. (**d**) Maximum z-projection of objects map for GFAP-positive astrocytes generated from a confocal z-stack (see Figure 2b). Individual astrocytes in the z-stack were identified by GFAP-specific fluorescence and converted to objects. Numbers indicate individual cell counts. n = 272 astrocytes in a volume of 400 µm × 400 µm × 59 µm. The object colors indicate different z-positions (darker colors in the back and brighter colors in the front). (**e**) Detail of area indicated by white box in Figure 3d. Green arrows indicate RABV-positive astrocytes. (**f**) Overlay of objects map (greyscale) with RABV P signals allows identification and counting of rRABV Fox-infected astrocytes.

Whereas immunofluorescence analysis in Figure 2 clearly demonstrated infection of astrocytes and neurons, they also revealed that only a fraction of both cell types was positive for RABV P. To quantify the ratio of infected and non-infected neurons and astrocytes, 3D object segmentation and counting was performed for NeuN- (Figure 3a,b) or GFAP (Figure 3d,e)-positive cells. The object map was merged with their respective RABV fluorescence (Figure 3c,f; Supplementary Video S3), and the number of RABV-positive neurons and astrocytes was determined by manual counting of RABV P- and cell marker-positive cells. For the z-stack shown in Figure 2b and Figure 3, total numbers of 762 neurons and 272 astrocytes were counted (Table 2, region 2), of which 16 and 18 were RABV positive, respectively.

Analysis of six z-stacks from different RABV-infected brain areas of the same sample led to the detection of 11,089 neurons and 2042 astrocytes, of which 3.9% and 7.0% were RABV positive, respectively (Table 2). The fraction of RABV-positive cells ranged from 2.1% to 6.1% (SD: ± 1.4) for neurons and 1.8% to 17.0% for astrocytes (SD: ± 4.9) (Table 2). These data indicated that rRABV Fox infects astrocytes and neurons in the mouse brain to comparable levels.

Observation of three different rRABV Fox-infected mice revealed that the infection level for astrocytes differed between single animals from 1.2% to 15.6% (SD: ± 6.7) (Supplementary Table S1). Nevertheless, in spite of some variance between individual mice and/or between different analyzed brain regions, detection of infected non-neuronal astrocytes in all animals at surprisingly high levels indicates that astrocyte infection by RABV has been underestimated thus far.

### 3.4. Astrocyte Infection by RABV Depends on the Type of Virus (Field vs. Lab-Adapted).

To compare the astrocyte tropism of field and lab-adapted viruses after i.m. inoculation, brains of mice infected with three different field viruses (rRABV Fox, rRABV Dog, and rRABV Rac) and two lab-adapted viruses (rCVS-11 and ERA) were analyzed. SAD L16 was excluded from these analyses since it is not able to induce clinical signs after i.m. inoculation (Supplementary Figure S3d).

Whereas rRABV Fox and rRABV Dog caused disease even after infection with a very low virus dose [42], infections with rRABV Rac, rCVS-11, and ERA did not show any clinical signs at a dose of 10^2^ TCID_50_ (Supplementary Figure S3). High dose infection with the ERA strain (10^5^ TCID_50_) led to 100% disease development (Supplementary Figure S3c), whereas high dose infections with rRABV Rac and rCVS-11 only caused disease in 50% and 16.7% of the infected mice, respectively (Supplementary Figure S3a,b). Accordingly, available tissue samples for the latter two viruses were limited to two and one infected brain. Imaging and quantification of at least six confocal z-stacks from different RABV-infected regions per infected brain was performed.

**Figure 4:**
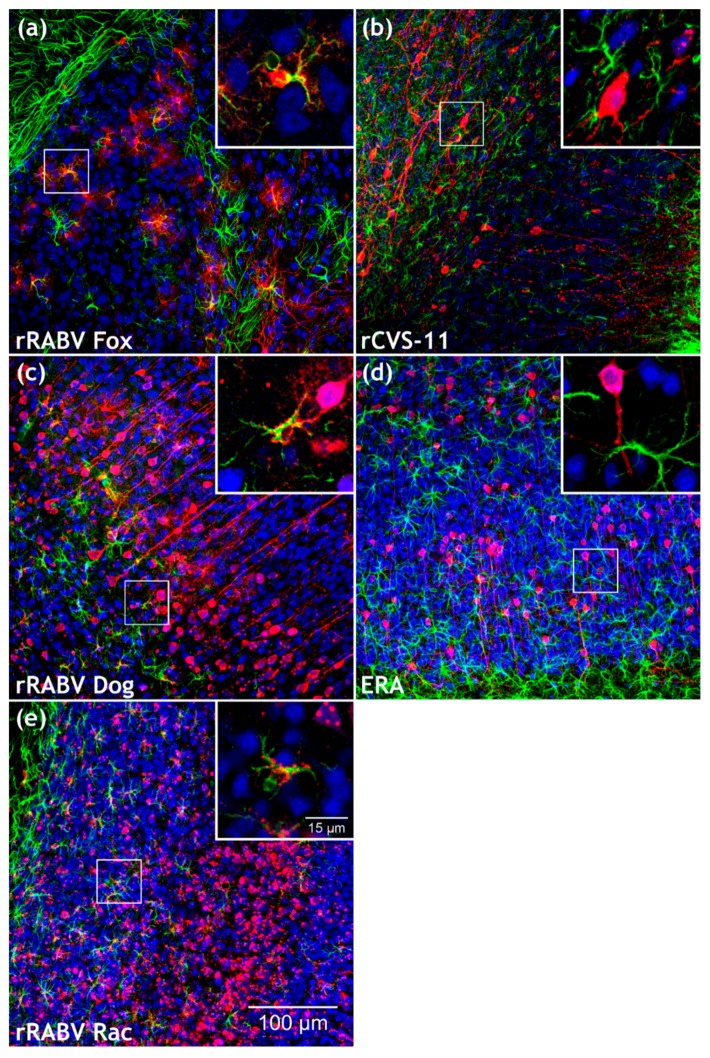
Comparison of field and lab RABV-infected brains after intramuscular (i.m.) infection with rRABV Fox, rRABV Dog, rRABV Rac, rCVS-11, and Evelyn Rokitnicki Abelseth (ERA). (**a–e**) Maximum z-projections of z-stacks [x, y = 400 µm, 400 µm (**a–e**); z = 59 µm (**a**, rRABV Fox), 66 µm (**b**, rCVS-11), 49 µm (**c**, rRABV Dog), 100 µm (**d**, ERA) and 89 µm (**e**, rRABV Rac)] after indirect immunofluorescence for RABV phosphoprotein P (red), GFAP (green), and NeuN (blue). To improve visualization of the maximum z-projections, some z-stacks were reduced in thickness. For the full z-stacks, refer to Supplementary Video S4. Insets in Figure 4a,c,e show RABV P accumulation (red) at GFAP-positive cells (green). Insets in Figure 4b,d show NeuN- (blue) and RABV P (red)-positive neurons. For the individual channels of the detail images, see Supplementary Figure S4.

RABV infections were readily detectable in the brain for all five viruses (Figure 4; Supplementary Video S4). Whereas RABV P antigen accumulated at GFAP-positive structures in rRABV Fox, rRABV Dog, and rRABV Rac-infected brains (Figure 4a,c,e), similar accumulations were not observed in rCVS-11 or ERA-infected brains, in which only infection of NeuN-positive neurons was detected (Figure 4b,d).

**Figure 5:**
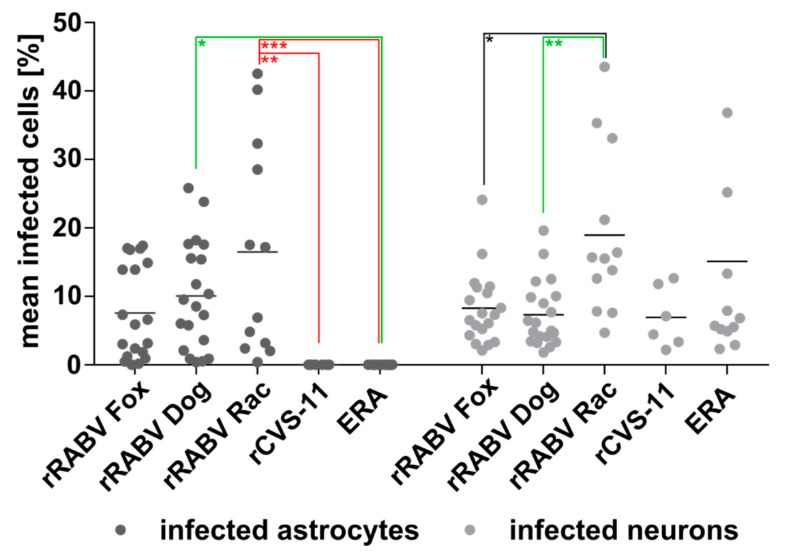
Percentage of field and lab RABV-infected neurons and astrocytes after i.m. inoculation. Per virus, 3 to 9 × 10^3^ astrocytes and 1.5 to 4 × 10^4^ neurons were counted in 19 (rRABV Fox), 20 (rRABV Dog), 12 (rRABV Rac and ERA), and six (rCVS-11) independent confocal z-stacks in three (rRABV Fox and rRABV Dog), two (rRABV RAC and ERA), and one (rCVS-11) animal (see Supplementary Table S1). Each dot represents the frequency of infected astrocytes or neurons in an analyzed z-stack. Mean values are provided as horizontal lines. * *p* ≤ .05; ** *p* ≤ .01; *** *p* ≤. 001 (two-way ANOVA with Tukey’s multiple comparison test).

Infections with rRABV Fox, rRABV Dog, rRABV Rac, rCVS-11, and ERA led to a mean of 8.3%, 7.3%, 18.9%, 6.9%, and 15.1% RABV-positive neurons (Figure 5; Supplementary Table S1), respectively, demonstrating a comparable level of neuron infection by all five viruses in clinically diseased mice. However, significant differences were observed for the astrocyte infections. Whereas rRABV Fox, rRABV Dog, and rRABV Rac infected 7.6% (SD: ± 6.7), 10.1% (SD: ± 7.7), and 16.5% (SD: ± 15.0) of the astrocytes, no RABV-positive astrocytes were detected in rCVS-11 and ERA-infected samples (Figure 5; Supplementary Table S1). These data indicated that, in contrast to the tested field viruses, the lab-adapted RABVs rCVS-11 and ERA did not infect astrocytes to detectable levels in vivo after i.m. inoculation.

### 3.5. Confirmation of the Specific Astrocyte Tropism of Field RABV, ERA, and SAD L16 after i.c. Inoculation and Route-Dependent Astrocyte Infection by rCVS-11

To test whether the inoculation route affects astrocyte infection and whether the highly attenuated SAD L16 virus is comparable to the SAD vaccine progenitor strain ERA, brain samples from two animals i.c.-infected with rRABV Fox, rRABV Dog, rCVS-11, or SAD L16 and one infected with the ERA strain were analyzed. 

**Figure 6:**
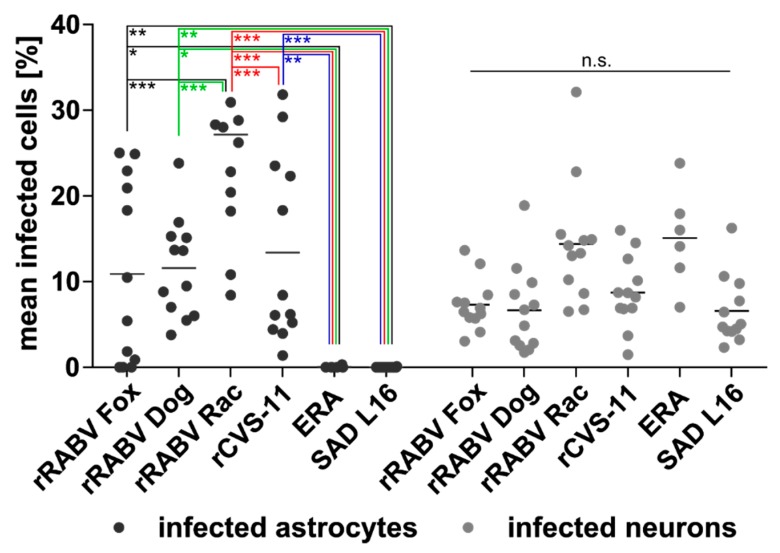
Percentage of field and lab RABV-infected neurons and astrocytes after i.c. inoculation. Per virus, 5 to 9 × 10^3^ astrocytes and 1.3 to 3 × 10^4^ neurons were counted in 12 (rRABV Dog, rRABV Fox, rRABV Rac, rCVS-11, and SAD L16) and six (ERA) independent confocal z-stacks in two (rRABV Fox, rRABV Dog, rRABV Rac, rCVS-11, and SAD L16) and one (ERA) animal (see Supplementary Table S2). Each dot represents the frequency of infected astrocytes or neurons in an analyzed z-stack. Mean values are provided as horizontal lines. * *p* ≤ .05; ** *p* ≤. 01; *** *p* ≤. 001; n.s. = not significant (two-way ANOVA with Tukey’s multiple comparison test).

**Figure 7:**
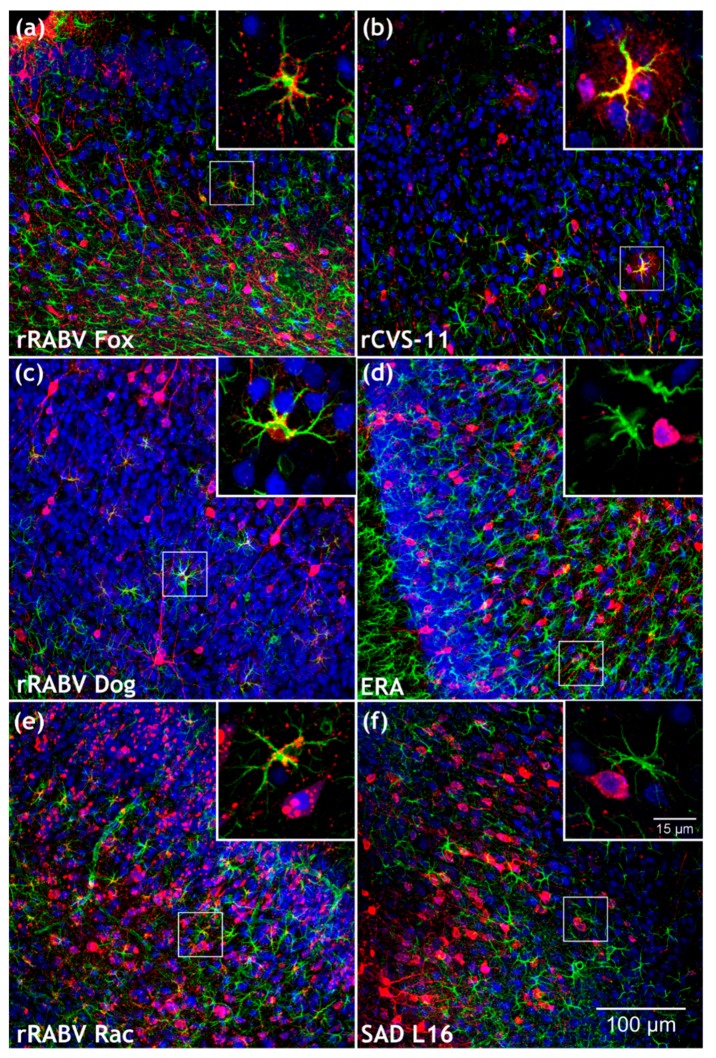
Comparison of field and lab RABV-infected brains after i.c. infection. (**a**–**f**) Maximum z-projections of z-stacks [x, y = 400 µm, 400 µm (**a**–**f**); z = 21 µm (**a**, rRABV Fox), 48 µm (**b**, rCVS-11), 74 µm (**c**, rRABV Dog), 98 µm (**d**, ERA), 75 µm (**e**, rRABV Rac) and 67 µm (**f**, SAD L16)] after indirect immunofluorescence for RABV phosphoprotein P (red), GFAP (green), and NeuN (blue). Insets in Figure 7a,b,c,e show RABV P accumulation (red) at GFAP-positive cells (green). To improve visualization of the maximum z-projections, some z-stacks were reduced in thickness. Insets in Figure 7d,f show NeuN- (blue) and RABV P (red)-positive neurons. For the individual channels of the detail images, see Supplementary Figure S5.

With astrocyte infections at frequencies of 10.9% (SD: ± 10.3), 11.6% (SD: ± 5.6), and 27.2% (SD: ± 12.8) (Figure 6; Supplementary Table S2), rRABV Fox, rRABV Dog, and rRABV Rac led to robust astrocyte infection via the i.c. route and thus confirmed their ability to establish astrocyte infection in vivo (Figure 7a,c,e). Lack of detectable astrocyte infection for the ERA and SAD L16 further confirmed that these viruses are not able to infect astrocytes to a detectable level (Figure 7d,f and Figure 6), even after direct virus administration into the brain.

Notably, in contrast to the i.m. inoculation route and to the other i.c.-inoculated lab-adapted RABVs SAD L16 and ERA, 13.4% (SD: ± 10.4) of the astrocytes were RABV-positive in rCVS-11-infected animals, indicating that, in the case of rCVS-11, the infection route has a substantial influence on astrocyte infection in the brain. However, the ratio of rCVS-11-infected neurons (8.7%; SD: ± 4.0) remained comparable to i.m. infections.

## 4. Discussion

By use of a novel immunofluorescence-compatible technique for 3D immunofluorescence imaging of solvent-cleared brain tissue slices [38,39] adapted to the imaging of RABV infections in brain tissue [40], we investigated the cell tropism of six different RABV isolates and lab-adapted strains. Both neurons and astrocytes display heterogeneous morphologies with pronounced three-dimensional projections. Compared to conventional thin sections or even in silico 3D reconstructions of serial thin sections, optical slicing of 1 mm-thick tissue samples during image acquisition allowed fast and seamless 3D reconstruction of immunostained tissues and high-resolution dissection of the detected antigens [40]. For the first time, this allowed systematic analysis and comparison of the tropism of the different viruses in large 3D volumes of infected mouse brains. One example for the superiority of the employed technique is the unambiguous detection of rCVS-11-infected astrocytes after i.c. inoculation (Figure 7 and Figure 6), whereas conventional thin layer immunohistochemistry analyses led to the assumption that infection of glial cells by CVS does not occur, even after i.c. inoculation [55].

Importantly, more than 10^4^ neurons and 10^3^ astrocytes (Supplementary Table S1 and S2) were quantitatively investigated for RABV infection in their authentic environment per virus and inoculation route. This provided reliable datasets about the frequencies of RABV detection in the two cellular subpopulations in vivo and statistically significant differences in astrocyte infections, which depended on the degree of virulence and the inoculation route of the tested RABVs (Figure 5 and Figure 6).

Because of the clonal and the defined nucleotide sequences, the recombinant viruses rRABV Fox and rRABV Dog [42], rRABV Rac (this work), SAD L16 [43], and rCVS-11 (this work) were selected for the analyses (full genome nucleotide and G protein amino acid sequence comparisons are provided in Supplementary Tables S3 and S4, and Supplementary Figure S6). Since SAD L16 was directly derived from the live vaccine SAD B19 vaccine strain [56], which is equally apathogenic after intramuscular inoculation, the non-recombinant SAD B19 progenitor vaccine virus strain ERA was included to allow for the investigation of attenuated live vaccine virus astrocyte tropism after peripheral i.m. inoculation. Compared to SAD B19, the ERA strain has a less intense cell culture passage history [45] and is still pathogenic in mice after peripheral inoculation [57]. Indeed, whereas SAD L16 was not able to cause disease after i.m. inoculation, ERA was even more pathogenic than rCVS-11 at the high i.m. inoculation dose of 10^5^ TCID_50_ with 100% and 16.7% of mice developing clinical signs, respectively (Supplementary Figure S3).

Both the progenitor field virus isolates and the respective recombinant virus clones rRABV Fox and rRABV Dog have been shown to share comparable pathogenicity with efficient disease development after i.m. infection of mice [42]. For the two aforementioned field virus isolates as well as for the raccoon RABV isolate used here for the generation of rRABV Rac, virulence in raccoons has been demonstrated [46]. Notably, although the raccoon virus isolate was highly virulent in its natural reservoir host, the recombinant clone rRABV Rac generated here was less virulent in mice, showing a pathogenicity of 50% only at the high i.m. inoculation dose (Supplementary Figure S3).

In striking contrast to rRABV Fox and rRABV Dog, the lab virus clone rCVS-11 was less efficient via the i.m. route but still able to cause disease in one out of six mice at a high virus dose of 10^5^ infectious units per mouse (Supplementary Figure S3). This perfectly matched previous studies, where the non-recombinant progenitor CVS-11 strain caused disease in only 16.7% of the mice at high i.m. dose inoculation, and, as observed here (Supplementary Figure S3), was apathogenic at low dose i.m. infection [51].

In contrast to the in vivo results, in vitro infection of mixed rat hippocampal neuron/astrocyte cultures with rRABV Fox, rRABV Dog, SAD L16, and rCVS-11 led to comparable levels of astrocyte infections, as demonstrated by GFAP-positive, RABV-infected cells (Figure 1) in a range from 4.9% to 6.7% for all viruses (Table 1). These data were in accordance with the general susceptibility of cultivated astrocytes for RABV infection repeatedly shown for field and lab-adapted viruses [29,34], with preferential replication in neurons [32]. Whereas Tsiang et al. reported 90–99% of the primary astrocytes free of RABV antigen, our results indicate that there is less variation within the tested viruses, with virus antigen detection in about 5% of the cultivated astrocytes independent of whether they were of field virus or lab strain origin (Table 1). Overall, comparison of the in vitro and the in vivo data showed that a general susceptibility of a primary CNS cell subpopulation in vitro (Figure 1) does not necessarily reflect the situation in vivo (Figure 2, Figure 4 and Figure 6).

Reasons for this could be altered antiviral response profiles in the more disordered cell culture conditions, different replication and spreading kinetics of the viruses in vivo, or differences in the non-synaptic release of infected neurons to allow infection of non-synaptically connected CNS cells. Whereas no information is available about the latter thus far, it is known that astrocytes are abortively infected by a chimeric SAD L16 virus expressing CVS-11 glycoprotein in mouse brains [33]. This supports the idea that replication of rCVS-11, ERA, and SAD L16 was similarly blocked in astrocytes by potent innate immune responses after infection via the i.m. or the i.c. inoculation routes, respectively. However, since neither viral genome copies nor virus mRNA levels were measured here, it remains to be clarified whether replication kinetics of the lab strains indeed differ from those of the field strains in the infected brains.

Nevertheless, it is conceivable that a more efficient replication of rRABV Fox, rRABV Dog, and rRABV Rac, and therefore the accumulation of the major interferon antagonist phosphoprotein P [8,9] in astrocytes, led to inhibition of antiviral responses. Indeed, lab-attenuated RABV has been shown to differ from wild-type RABV by the induction of an increased type I interferon (IFN) production and expression of inflammatory cytokines via the mitochondrial antiviral-signaling protein (MAVS) pathway [34]. Accordingly, the immunofluorescence detection of RABV P for the identification of infected cells (Figure 2, Figure 4 and Figure 6) confirmed the presence of abundant levels of the major interferon antagonist in the field virus-infected astrocytes. It is highly unlikely that the lack of P detection in the astrocytes of lab RABV-infected animals was due to a major difference in P gene expression of field viruses, since P was readily detectable in lab RABV-infected neurons.

Besides supporting virus replication in astrocytes by decreasing release of inflammatory cytokines, robust field virus replication in these cells may result in a less pronounced or delayed general antiviral response to field viruses. This would be in accordance with the observed immune escape of field viruses in infected animals [35] and may represent a major immunological difference to lab-adapted strains. However, further experimentation will be needed to test this hypothesis.

Notably, and in contrast to the ERA strain not being detected in astrocytes after i.c. inoculation, the astrocyte infection by rCVS-11 was at a frequency of 13.4% after i.c. inoculation (Figure 6 and Supplementary Table S2) and also at higher levels than observed for the field viruses rRABV Fox and rRABV Dog after both i.c. and i.m. infection. This revealed that the ability for rCVS-11 to establish an infection in these cell types depended on the infection route. Since infection from the periphery relies on trans-synaptic spread of the viruses and the virus may not become visible for immune and non-neuronal target cells, astrocyte infection could represent a late phase phenotype of brain infection, where abundant infection of neurons and non-synaptic release of virus particles may facilitate astrocyte infection. Consequently, astrocyte-mediated immune reactions would be delayed and virus elimination prior to disease onset not possible. Compared to the field viruses, slower virus replication and/or spreading kinetics of rCVS-11 could lead to lower levels or the absence of detectable astrocyte infection after i.m. inoculation (Figure 5), although the virus is principally able to infect these cell types (Figure 7b). Indeed, the efficacy of trans-synaptic retrograde spread can differ between a highly neurotropic CVS-24 virus variant and an SAD L16-like vaccine virus [24]. On a higher level, this may also distinguish highly virulent field viruses such as rRABV Fox and rRABV Dog from rCVS-11. Furthermore, it is conceivable that i.c. inoculations represent a shortcut to brain infection with simultaneous and multiple infection of neurons and astrocytes in a non-transsynaptic manner. Higher numbers of infected cells at the beginning of CNS infection compared to trans-synaptic invasion after i.m. infection may lead to faster virus spread and infection of multiple regions of the brain with abundant late phase virus release and astrocyte infection, similar to that speculated above for i.m. infections with highly virulent field viruses.

Indeed, astrocyte activation by other viruses have been shown to occur earlier after i.c. than after peripheral infections [58]. Both virus and cell response kinetics may differ between the two infection routes. Increased induction of neuronal cell death after i.c. RABV infection compared to no detectable apoptosis in i.m.-infected animals [59] further indicates qualitative differences in host reaction to the virus. The special role of astrocytes in the CNS as a main source of IFN-β expression and virus control through TLR (Toll-like receptor) and RLR (RIG-I-like receptor) activation pathways [33,60,61] in combination with the infection route-dependent differences described here, as observed for rCVS-11, may contribute to such differences in apoptosis and other host reaction patterns. However, further studies must clarify whether different infectious route-dependent and -independent virus kinetics can determine astrocyte tropism in vivo and how this affects downstream host reactions. Since differences in innate immune induction through dsRNA between field and attenuated viruses were demonstrated [34], the innate immune induction potential of rRABV Fox, rRABV Dog, rRABV Rac, rCVS-11, ERA, and SAD L16 in astrocytes has to be investigated in order to assess whether the route dependency of rCVS-11 is also affected by different levels of innate immune induction.

Most likely, lack of astrocyte infection by SAD L16 and ERA was the outcome of strong virus inhibition and elimination, as abortive astrocyte infection by a comparable virus has previously been suggested [33]. Whether less antiviral response induction may allow the more virulent rCVS-11 or the highly virulent field viruses to overcome a threshold of virus replication and antagonist expression—and thus may support further replication—will be addressed in future studies. Even though the underlying mechanisms cannot be clarified here, comparable results for SAD L16 and the still pathogenic ERA strain strongly suggest that a general block of detectable astrocyte replication of these vaccine strains represents a major difference to the other tested viruses.

Only by using novel 3D immunofluorescence techniques, we were able to provide comprehensive analyses of the cell tropism of highly virulent field RABVs and less virulent or attenuated lab strains. In particular, high-resolution 3D images and quantitative downstream analysis provided novel insights in the infection processes at the clinical phase of this deadly disease. Although independent of detectable astrocyte infection, symptoms and lethal progression of the disease occur once the virus efficiently spreads in the brain after infection with all six tested viruses (Figure 7 and Supplementary Figure S3). However, different virus kinetics and astrocyte-related innate immune reactions may affect the progression kinetics, immune pathogenicity, and further spread of the virus to peripheral salivary glands. The latter may represent a key issue in terms of field virus transmission and maintenance in host populations.

Whereas these aspects must be addressed in separate trials, this study provides a novel and quantitative basis for a new, dynamic view on RABV host interactions in vivo on the cellular level. Also, this approach showcases the potential of immunostaining-compatible tissue clearing/3D imaging techniques to specifically investigate virus–host interactions at high-resolution on cellular and subcellular levels. While this study focused on standard cellular markers for neurons and astrocytes, future approaches including immune and host pathway markers will pave the way for direct high-resolution imaging-based analysis of infection processes in complex and morphologically preserved tissues.

## Figures and Tables

**Table 1:** Infection of GFAP- and MAP2-expressing cells in hippocampus cell cultures. RABV-infected cells were counted after indirect immunofluorescence detection of RABV N protein and subdivided in cells that were either negative (−) or positive (+) for GFAP/MAP2. Because of overlaying positive and negative cells in GFAP and MAP2 stainings, a clear assignment was not always possible. Such cells were classified as uncertain. CVS: Challenge Virus Street.

Virus	GFAP (−)		GFAP (+)		Uncertain	
	Count	%	Count	%	Count	%
SAD L16	256	48.3	30	5.7	244	46
rCVS-11	58	64.4	6	6.7	26	28.9
rRABV Dog	252	46.8	29	5.4	258	47.9
rRABV Fox	1002	55.2	89	4.9	712	39.5
**Virus**	**MAP2 (−)**		**MAP2 (+)**		**Uncertain**	
	**Count**	**%**	**Count**	**%**	**Count**	**%**
SAD L16	18	3.5	387	74.2	116	22.3
rCVS-11	1	1.0	44	43.2	57	55.9
rRABV Dog	27	5.3	347	73.3	109	21.4
rRABV Fox	190	7.3	2096	80.3	324	12.4

**Table 2:** Quantification of rRABV Fox-infected neurons and astrocytes. Results of the analysis of six different areas of one infected mouse brain. The quantification pipeline of region 2 is depicted in Figure 3. Neurons and astrocytes were segmented, automatically counted, and overlaid with the respective RABV P signals. Infected cells were counted manually.

	Neurons					Astrocytes				
Region	Counted	Infected	%	Mean	SD	Counted	Infected	%	Mean	SD
1	485	21	4.3	3.9	1.4	362	11	3.0	7.0	4.9
2	762	16	2.1			272	18	6.6		
3	448	13	2.9			106	18	17.0		
4	2518	132	5.2			488	9	1.8		
5	3029	91	3.0			273	20	7.3		
6	3847	234	6.1			541	32	5.9		
∑	11098	507				2042	108			

## Supplementary Materials

The following are available online at https://www.mdpi.com/2073-4409/9/2/412/s1, Captions_SupplementaryFiles: Captions and legends to supplementary figures, tables, and videos. Figure S1: Immunofluorescence of non-infected astrocytes and neurons in vitro. Figure S2: Details of rRABV Fox-infected, NeuN-positive neurons. Figure S3: Kaplan-Meyer survival plots for rRABV Rac, rCVS-11, ERA, and SAD L16. Figure S4: Details of field virus (rRABV Fox, rRABV Dog, and rRABV Rac) and lab RABV (rCVS-11 and ERA) infections after i.m. inoculation. Figure S5: Details of field virus (rRABV Fox, rRABV Dog, and rRABV Rac) and lab RABV (rCVS-11, ERA, and SAD L16) infections after i.c. inoculation. Figure S6. Amino acid alignment of glycoprotein G of rRABV Rac, rCVS-11, SAD L16, rRABV Fox, and rRABV Dog. Table S1: Quantification of RABV-infected neurons and astrocytes after i.m. infection. Table S2: Quantification of RABV-infected neurons and astrocytes after i.c. infection. Table S3. Nucleotide sequence alignment of full genome sequences of rRABV Rac, rCVS-11, SAD L16, rRABV Dog, and rRABV Fox. Table S4. Alignment of G protein amino acid sequence of rRABV Rac, rCVS-11, SAD L16, rRABV Dog, and rRABV Fox. Video S1: 3D projections of rRABV Fox-infected astrocytes and neurons in two different areas of a mouse brain. Video S2: 3D projection of an rRABV Fox-infected astrocyte in a mouse brain. Video S3: 3D projection of the quantification of RABV-infected neurons and astrocytes. Video S4: 3D projections of field and lab RABV-infected brains after i.m. infection with rRABV Fox, rRABV Dog, rRABV Rac, rCVS-11, and ERA, and SAD L16 after i.c. infection.
