# Supplementary material for: Astrocyte Infection during Rabies Encephalitis Depends on the Virus Strain and Infection Route as Demonstrated by Novel Quantitative 3D Analysis of Cell Tropism
Source: Cells. 2020 Feb 11;9(2):412. doi: 10.3390/cells9020412 (PMC7072253; doi:10.3390/cells9020412)
Supplement: Supplementary file 1 [file cells-09-00412-s001.zip › Captions_SupplementaryFiles.pdf]

cells-707879 Captions Supplementary Files

Astrocyte infection during rabies encephalitis depends on the virus strain and infection route as demonstrated by novel quantitative 3D analysis of cell tropism

Madlin Potratz, Luca Zaeck, Michael Christen, Verena te Kamp, Antonia Klein, Tobias Nolden, Conrad M. Freuling, Thomas Müller and Stefan Finke

Figure S1: Immunofluorescence of non-infected astrocytes and neurons *in vitro*.

Figure S2: Details of rRABV Fox-infected, NeuN-positive neurons.

Figure S3: Kaplan-Meyer survival plots for rRABV Rac, rCVS-11, ERA, and SAD L16.

Figure S4: Details of street virus (rRABV Fox, rRABV Dog, and rRABV Rac) and lab RABV (rCVS-11 and ERA) infections after i.m. inoculation.

Figure S5: Details of street virus (rRABV Fox, rRABV Dog, and rRABV Rac) and lab RABV (rCVS-11, ERA, and SAD L16) infections after i.c. inoculation.

Figure S6. Amino acid alignment of glycoprotein G of rRABV Rac, rCVS-11, SAD L16, rRABV Fox, and rRABV Dog. Dissimilarities are highlighted by colors. Alignment and figure creation was performed with Geneious version 11.1 by Biomatters. Available from https://www.geneious.com.

Table S1: Quantification of RABV-infected neurons and astrocytes after i.m. infection. Results of the analysis of six to eight different regions of each infected mouse brain for three (rRABV Fox, rRABV Dog), two (rRABV Rac and ERA), and one (rCVS-11) animal infected via the i.m. route. Neurons and astrocytes were segmented, automatically counted, and overlaid with the respective RABV P signals. Infected cells were counted manually.

Table S2: Quantification of RABV-infected neurons and astrocytes after i.c. infection. Results of the analysis of six different regions of each infected mouse brain for two (rRABV Fox, rRABV Dog, rRABV Rac, rCVS-11, and SAD L16) and one (ERA) animal infected via the i.c. route. Neurons and astrocytes were segmented, automatically counted, and overlaid with the respective RABV P signals. Infected cells were counted manually.

Table S3. Nucleotide sequence alignment of full genome sequences of rRABV Rac, rCVS-11, SAD L16, rRABV Dog, and rRABV Fox. Shown are nucleotide identities of the respective viruses of which the cloned full-length cDNA copies have been sequenced. The SAD L16 precursor virus ERA (FLI ID N° 12829) was excluded from the comparison as the full genome sequence has not yet been determined for this ERA virus batch.

Table S4. Alignment of G protein amino acid sequence of rRABV Rac, rCVS-11, SAD L16, rRABV Dog, and rRABV Fox. Shown are amino acid identities of the respective viruses.

Video S1: 3D projections of rRABV Fox-infected astrocytes and neurons in two different areas of a mouse brain. 3D projections of z-stacks from Figure 2b and c (x, y, z = 400 µm, 400 µm, 59 µm for (**a**) and 400 µm, 400 µm, 103 µm for (**b**), respectively) after indirect immunofluorescence for RABV phosphoprotein P (red), GFAP (green), and NeuN (blue).

Video S2: 3D projection of an rRABV Fox-infected astrocyte in a mouse brain. (**a**) 3D projection of the detail view from Figure 2f with a GFAP-positive cell (green, (**c**)) and associated accumulation of RABV P (red, (**b**)). (**d**) No neuronal NeuN (blue) was detected.

Video S3: 3D projection of the quantification of RABV-infected neurons and astrocytes. (**a**) 3D projection of the objects map for NeuN-positive neurons from Figure 3c generated from a confocal z-stack (see Figure 2b). Overlay of objects map (greyscale) with RABV P signals allows identification and counting of rRABV Fox-infected neurons. (**b**) 3D projection of the objects map for GFAP-positive astrocytes from Figure 3f generated from a confocal z-stack (see Figure 2b). Overlay of objects map (greyscale) with RABV P signals allows identification and counting of rRABV Fox-infected astrocytes.

Video S4: 3D projections of street and lab RABV-infected brains after i.m. infection with rRABV Fox, rRABV Dog, rRABV Rac, rCVS-11, ERA, and SAD L16 after i.c. infection. (**a-f**) 3D projections of z-stacks (x, y = 400 µm, 400 µm (**a-f**); z = 59 µm (**a**, rRABV Fox), 66 µm (**b**, rCVS-11), 77 µm (**c**, rRABV Dog), 100 µm (**d**, ERA), 89 µm (**e**, rRABV Rac), and 67 µm (**f**, SAD L16)) after indirect immunofluorescence for RABV phosphoprotein P (red), GFAP (green), and NeuN (blue).
