# Supplementary figures and images for: Astrocyte Infection during Rabies Encephalitis Depends on the Virus Strain and Infection Route as Demonstrated by Novel Quantitative 3D Analysis of Cell Tropism
Source: Cells. 2020 Feb 11;9(2):412. doi: 10.3390/cells9020412 (PMC7072253; doi:10.3390/cells9020412)

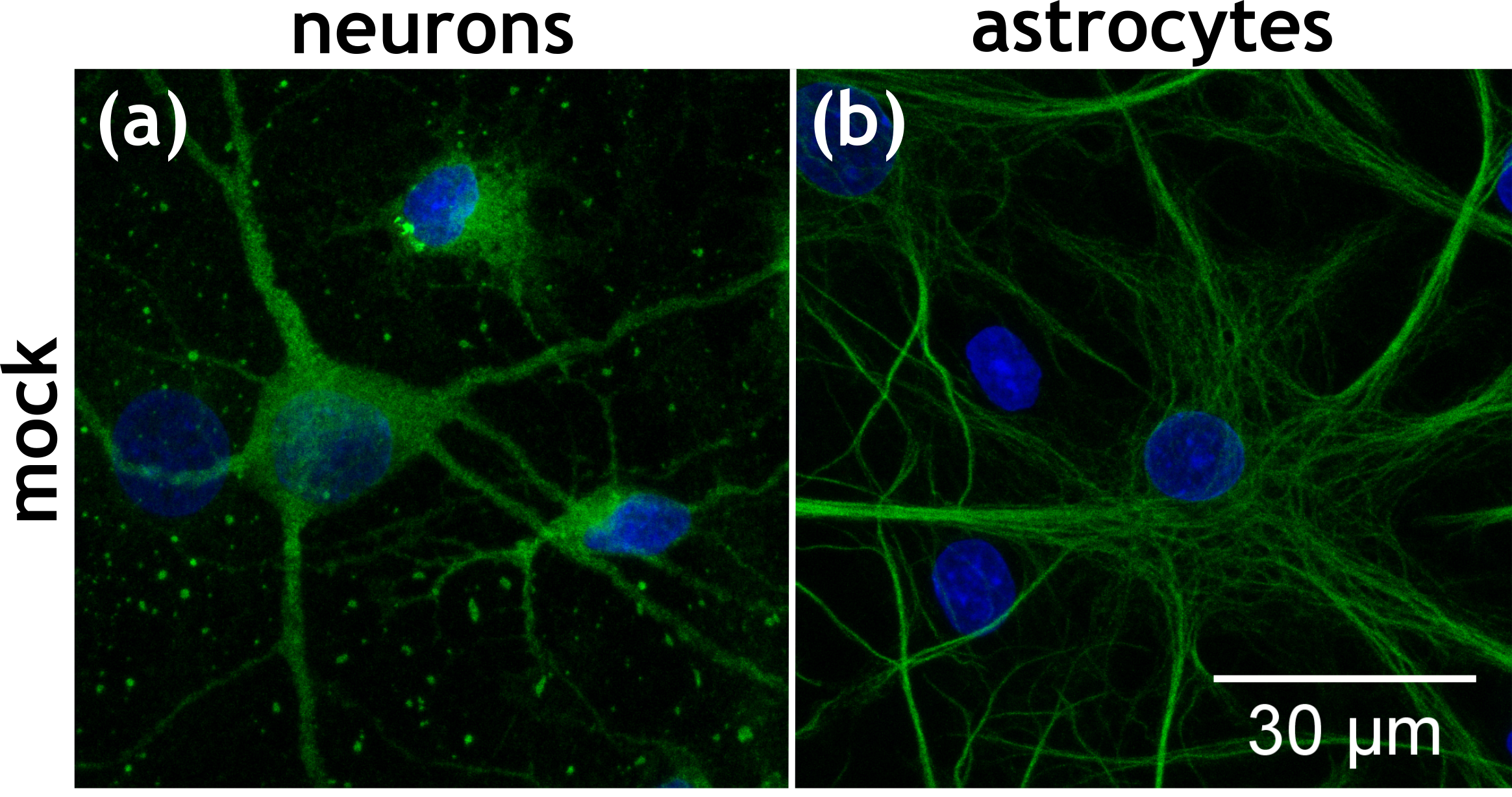

Supplement: Supplementary file 1 [file cells-09-00412-s001.zip › Figure S1.png]

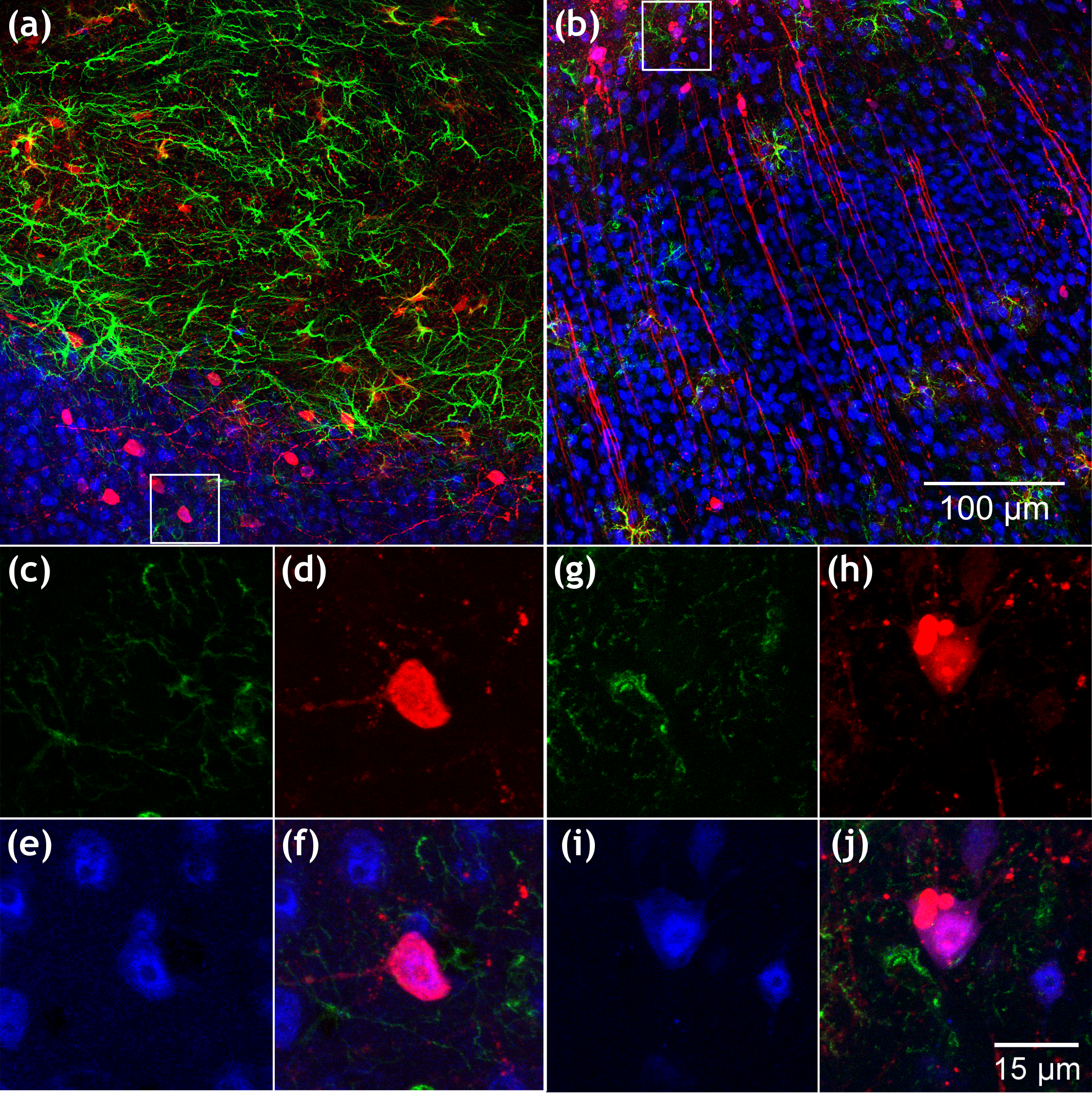

Supplement: Supplementary file 1 [file cells-09-00412-s001.zip › Figure S2.png]

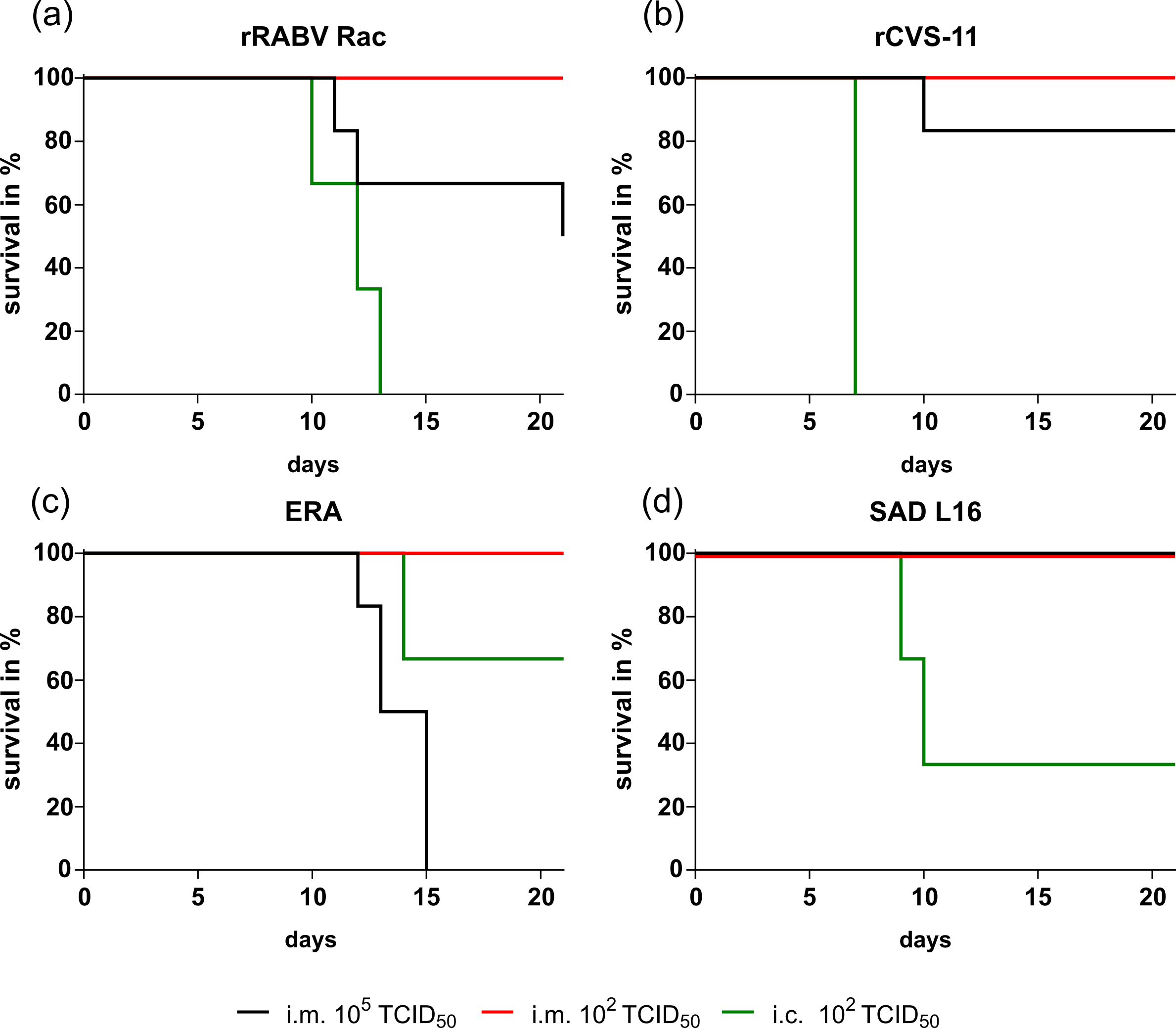

Supplement: Supplementary file 1 [file cells-09-00412-s001.zip › Figure S3.png]

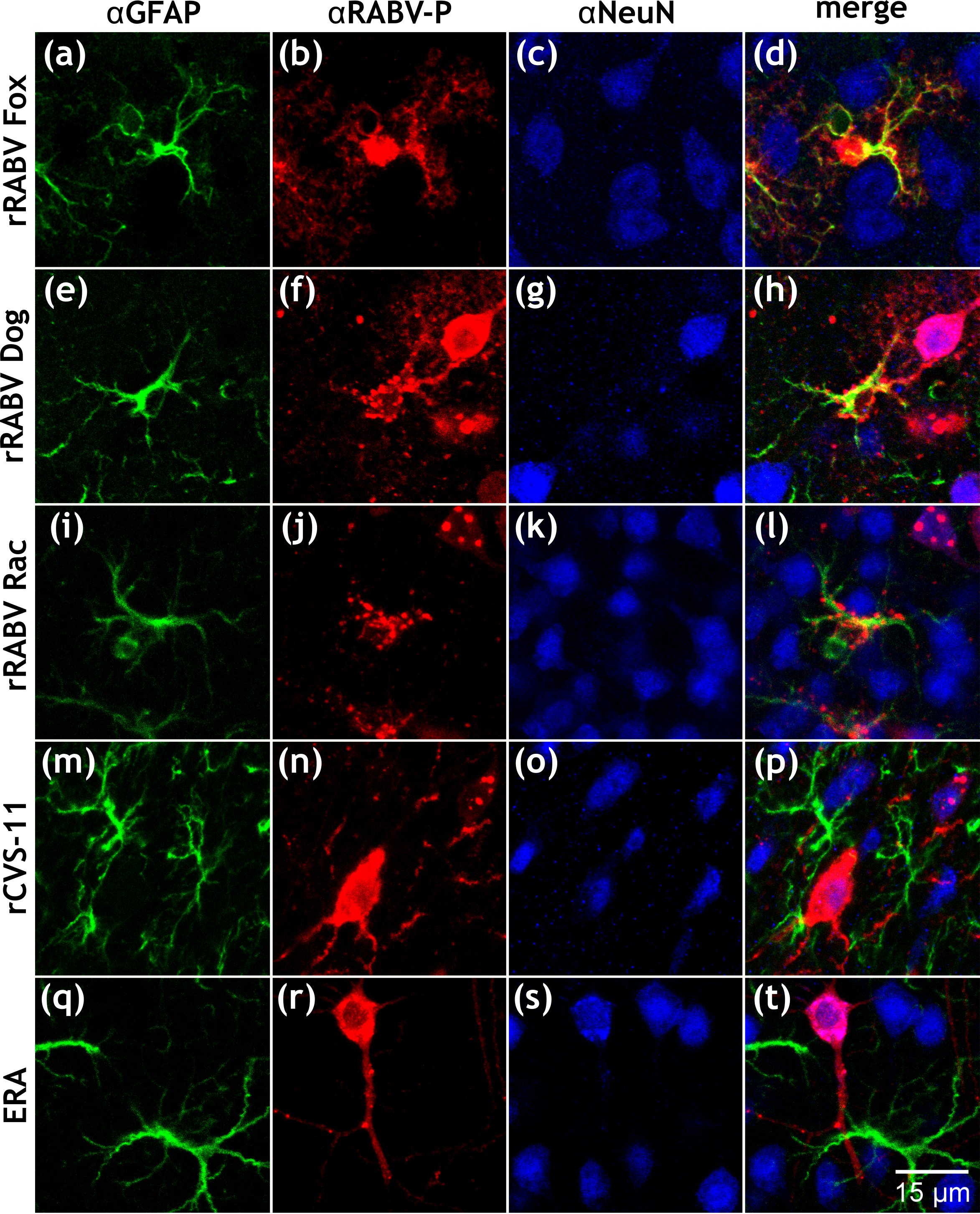

Supplement: Supplementary file 1 [file cells-09-00412-s001.zip › Figure S4.png]

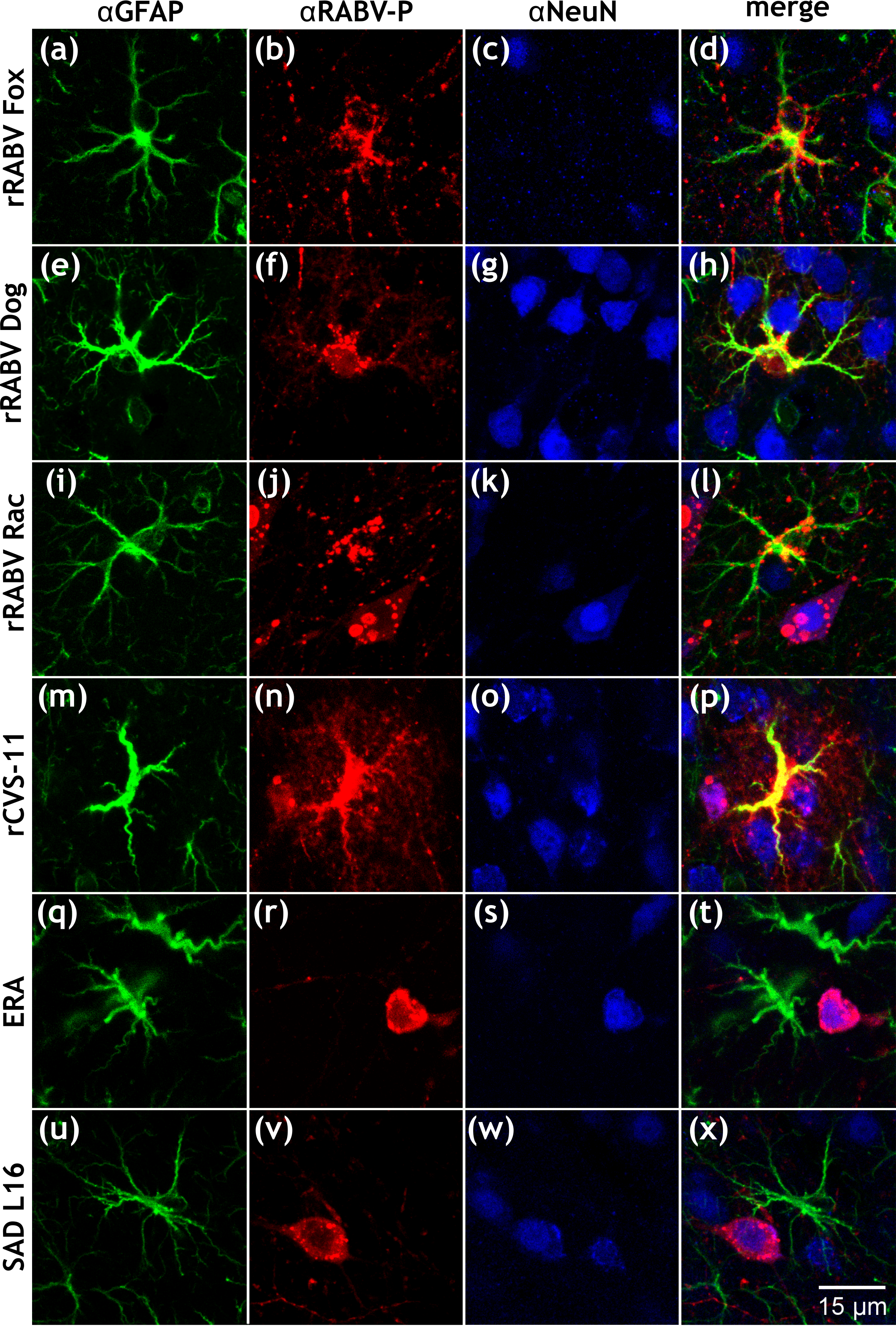

Supplement: Supplementary file 1 [file cells-09-00412-s001.zip › Figure S5.png]

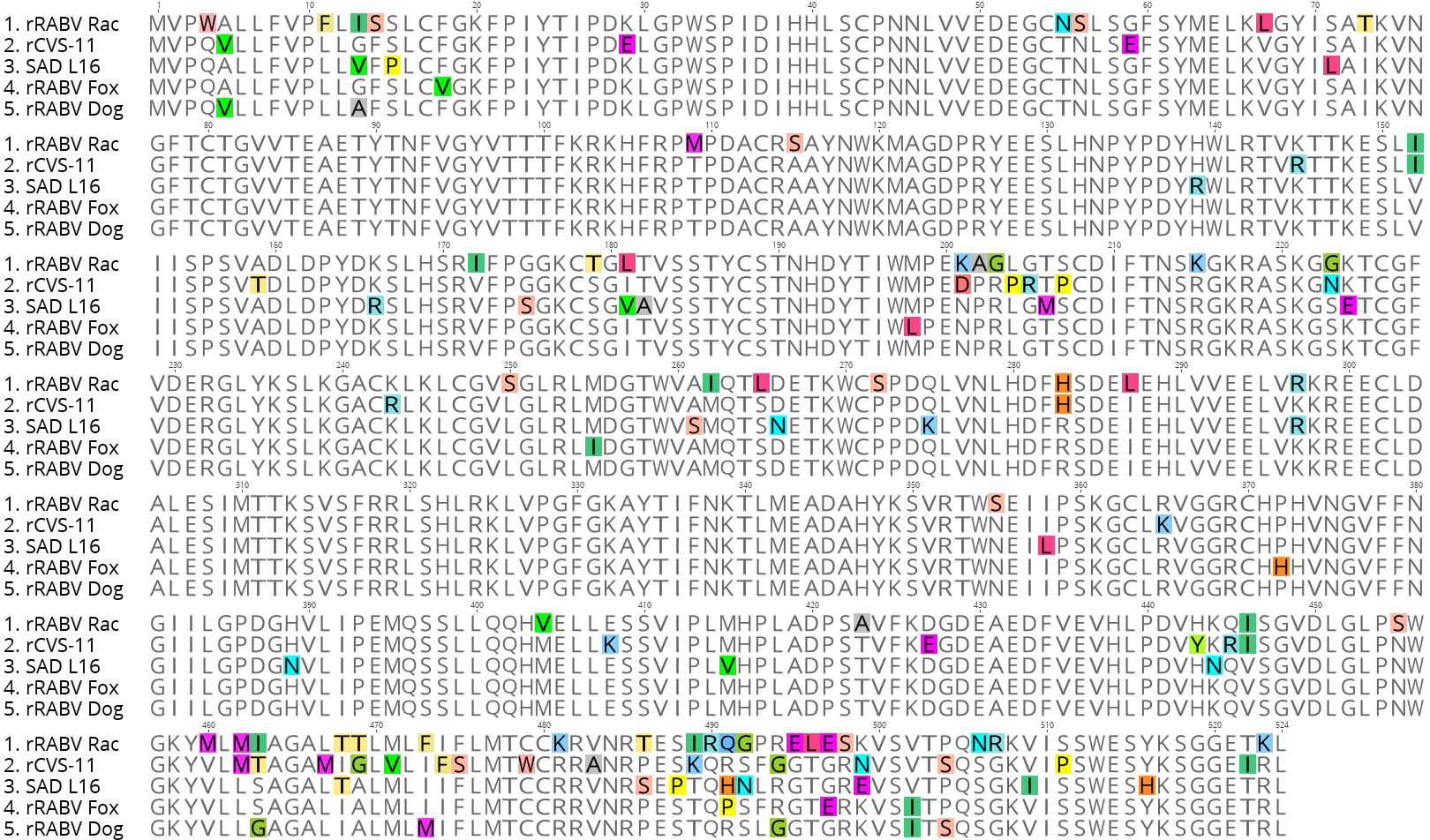

Supplement: Supplementary file 1 [file cells-09-00412-s001.zip › Figure S6.png]
